# Artificial Intelligence in Pediatric Dentistry: Current Applications, Emerging Trends, and Future Directions

**DOI:** 10.3390/dj14080493

**Published:** 2026-08-06

**Authors:** Omar A. El Meligy, Ahmed O. Elmeligy

**Affiliations:** 1 Pediatric Dentistry and Dental Public Health Department, Faculty of Dentistry, Alexandria University, Alexandria 21131, Egypt; 2 Department of Electrical and Computer Engineering, McGill University, Montreal, QC H3A 0G4, Canada; ahmed.elmeligy@mail.mcgill.ca

**Keywords:** artificial intelligence, pediatric dentistry, machine learning, deep learning, dental caries, diagnostic imaging, explainable artificial intelligence

## Abstract

**Background/Objective:** Artificial intelligence (AI) is increasingly transforming healthcare and has emerged as a promising tool in pediatric dentistry. This narrative review examines the methodological foundations, current applications, limitations, and future directions of AI in pediatric dental practice. **Methods:** A structured literature search was conducted in PubMed/MEDLINE, Scopus, Web of Science, and Google Scholar for relevant English-language publications from database inception through to May 2026, using predefined search terms and eligibility criteria. Following screening and full-text assessment, 72 unique publications were retained for the narrative synthesis. **Results:** AI applications in pediatric dentistry include early childhood caries detection and risk prediction, dental plaque assessment, identification of mesiodens and supernumerary teeth, dental age estimation, automated tooth detection, fissure-sealant evaluation, and craniofacial growth prediction. Several imaging-based models demonstrated performance comparable to experienced clinicians. However, many studies relied on retrospective, single-center datasets and lacked external or prospective validation. Additional concerns included algorithmic bias, data privacy, limited interpretability, and infrastructure requirements. **Conclusions:** AI has considerable potential to improve diagnostic efficiency, consistency, and personalized preventive care in pediatric dentistry. However, it should be used as a clinical decision-support tool rather than a replacement for professional judgment. Future research should prioritize prospective multicenter validation, multimodal and explainable systems, standardized reporting, and ethical clinical implementation.

## 1. Introduction

Artificial intelligence (AI) emerged as a formal field through the Dartmouth Summer Research Project on Artificial Intelligence, proposed in 1955 and held in 1956 [1]. Since then, AI has developed into a broad discipline encompassing computational systems capable of performing tasks associated with human intelligence, including learning from data, recognizing patterns, solving complex problems, and supporting decision-making [2,3,4,5]. Over the past decade, the integration of AI into healthcare and dentistry has accelerated substantially, driven by advances in computational power, the growing availability of clinical data, and the development of increasingly sophisticated machine learning (ML) and deep learning (DL) methods [6,7,8,9,10]. To maintain clinical relevance, the following discussion focuses on the practical significance of these approaches for pediatric dental diagnosis, decision-making, and patient care rather than on detailed algorithmic engineering.

Modern AI systems are largely driven by ML, a branch of AI that enables computational models to identify patterns and relationships directly from data. Unlike traditional rule-based systems, ML and DL models can detect subtle features that may not be readily apparent to clinicians. In dentistry, these data-driven approaches are increasingly applied to diagnostic imaging, risk prediction, treatment planning, and workflow optimization. By supporting the analysis of complex clinical and radiographic information, AI has the potential to improve diagnostic accuracy, enhance efficiency, and reduce variability in clinical practice.

In dentistry, AI has demonstrated considerable potential, particularly due to the specialty’s strong reliance on imaging modalities such as radiographs, cone-beam computed tomography (CBCT), and intraoral photography [8]. Deep learning models, especially convolutional neural networks (CNNs), have shown remarkable capabilities for detecting subtle patterns in dental images, enabling early identification of pathological conditions that may be overlooked during conventional examination [11,12,13,14,15,16]. Consequently, AI has been increasingly applied in areas such as caries detection, periodontal assessment, orthodontic analysis, and treatment planning.

Pediatric dentistry represents a particularly promising field for AI integration, given its emphasis on early diagnosis, preventive care, and growth monitoring. The dynamic nature of the developing dentition, combined with the need for timely intervention, makes accurate and efficient diagnostic tools essential. Recent studies demonstrate a wide range of AI applications in pediatric dentistry, including automated detection of dental plaque on primary teeth [17], early childhood caries prediction [18,19], identification of mesiodens and supernumerary teeth, assessment of craniofacial growth patterns, reduction in diagnostic variability, and prediction of oral health risks based on behavioral and environmental factors [20,21,22,23,24,25,26,27,28,29]. Furthermore, AI-driven tools may support personalized treatment strategies by integrating clinical, radiographic, demographic, and behavioral data.

Despite these advancements, several challenges hinder the widespread clinical adoption of AI in pediatric dentistry. Many existing models are trained on retrospective datasets or evaluated under highly controlled experimental conditions, reducing their generalizability and real-world reliability [20,30]. High-quality datasets that adequately represent diverse pediatric populations remain limited, increasing the risk of algorithmic bias and raising concerns about whether AI tools can perform fairly and consistently across different demographic groups [31,32]. In addition, most current systems rely heavily on imaging data, with insufficient integration of clinical examination findings, patient history, and behavioral determinants that are essential in pediatric care.

Concerns related to data privacy, algorithmic transparency, regulatory frameworks, and the “black box” nature of many deep learning models further complicate clinical implementation [6,30]. The development of explainable and interpretable AI (XAI) offers a promising solution by making model reasoning more transparent, improving clinician trust, and supporting safer decision-making [33,34]. However, successful deployment also requires robust digital infrastructure, secure data management systems, and ongoing technical support. Many dental clinics may lack the necessary IT resources and trained support staff to maintain AI systems, ensure cybersecurity, and integrate these tools effectively into existing workflows [35].

Therefore, while AI holds substantial promise as an adjunct tool for improving diagnostic accuracy and clinical decision-making in pediatric dentistry, its translation into everyday practice remains incomplete. A comprehensive and critical evaluation of current evidence is necessary to identify existing gaps, assess the robustness of available models, and guide future research toward clinically applicable and ethically sound AI solutions.

Accordingly, this article is presented as a narrative review and is organized thematically to synthesize the methodological foundations, advantages, limitations, clinical applications, and future directions of AI in pediatric dentistry.

## 2. Review Methodology

This narrative review was informed by a structured literature search conducted in PubMed/MEDLINE, Scopus, Web of Science, and Google Scholar. The search included English-language publications available from database inception through May 2026. Search terms related to artificial intelligence and pediatric dentistry were combined using the Boolean operators “AND” and “OR.” The principal search terms included “artificial intelligence,” “machine learning,” “deep learning,” “convolutional neural network,” “pediatric dentistry,” “paediatric dentistry,” “children,” “early childhood caries,” “dental plaque,” “mesiodens,” “supernumerary teeth,” “dental age estimation,” and “tooth detection.” The reference lists of relevant publications were also examined to identify additional eligible sources.

Publications were considered eligible if they addressed the development, validation, evaluation, or clinical application of AI-based methods relevant to pediatric dentistry. Original research articles, relevant review articles, and selected methodological publications were included. Studies were excluded if they focused exclusively on adult populations without clear pediatric relevance, were unrelated to dentistry or oral healthcare, represented duplicate publications, were conference abstracts lacking sufficient methodological detail, or had no accessible full text. Titles and abstracts were initially screened, followed by full-text assessment of potentially relevant publications. Following this process, 72 unique publications were retained and cited in the final narrative synthesis. Because the review was narrative in design and a prospective screening log was not maintained, the exact numbers of records retrieved, excluded, and assessed at each stage could not be reliably reconstructed. Accordingly, no formal risk-of-bias assessment or quantitative meta-analysis was performed.

## 3. Foundations and Classification of Artificial Intelligence in Dentistry

AI in dentistry encompasses a spectrum of computational methods that analyze clinical, radiographic, and behavioral data to support diagnosis, prediction, and clinical decision-making [36,37]. These methods range from traditional ML models that rely on engineered features to advanced DL architectures capable of autonomously extracting patterns from raw data [38]. Understanding the distinctions among these approaches is essential for evaluating their capabilities, limitations, and suitability for different dental applications [39]. The following subsections outline the major categories of AI techniques used in dentistry, with emphasis on their methodological foundations and clinical relevance.

### 3.1. Machine Learning (ML)

Today, ML represents one of the foundational pillars of modern AI and is built upon principles of statistical inference and mathematical modeling [40,41]. ML algorithms are designed to detect relationships, trends, and latent structures within large volumes of data, enabling systems to process heterogeneous inputs, extract clinically relevant features, and generate predictions that support clinical decision-making [40]. In dentistry, ML is widely applied to tasks such as disease classification, risk assessment, and outcome prediction [40,42]. Classical algorithms, including logistic regression, support vector machines, random forests, and other ensemble methods, have been widely used to analyze clinical, demographic, and behavioral data [40,42]. These approaches are particularly valuable in pediatric dentistry, where they help identify risk factors for early childhood caries and other oral health conditions, facilitating early intervention and preventive care strategies [21,22,23,24].

Machine learning models learn patterns from previously collected clinical data and apply these patterns to new patients. In pediatric dentistry, they may assist with caries-risk prediction, disease classification, and treatment planning by combining clinical, behavioral, demographic, and radiographic information. Their clinical value depends on the quality and representativeness of the training data and on validation in populations different from those used during model development [41,42,43,44,45,46,47].

### 3.2. Deep Learning (DL)

Deep learning is an advanced form of machine learning that can automatically identify complex patterns directly from large datasets [42,48]. Unlike conventional approaches that depend heavily on manually selected features, deep-learning models can learn relevant information from raw data, particularly radiographs and clinical photographs [40,43].

**Figure 1:**
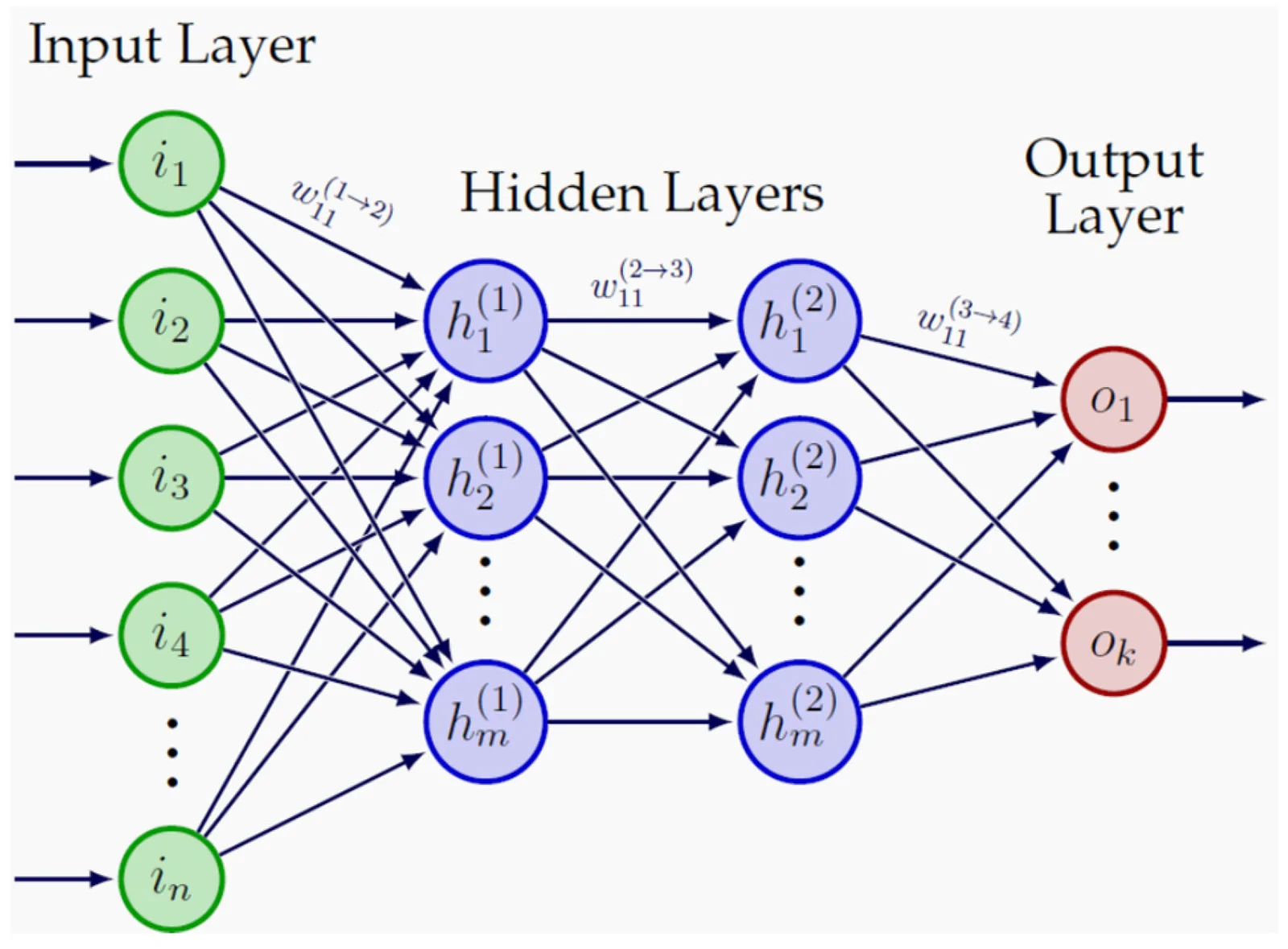
Simplified schematic representation of an artificial neural network consisting of an input layer, two intermediate hidden layers, and an output layer. The network progressively processes input data to generate a final prediction.

Artificial neural networks form the basis of deep learning and generally consist of an input layer, one or more intermediate hidden layers, and an output layer, as illustrated in Figure 1. These layers progressively process the input data to generate a prediction. In pediatric dentistry, deep-learning models may support caries detection, tooth identification, anatomical segmentation, dental-age assessment, and recognition of developmental anomalies.

Despite their clinical potential, deep-learning models have important limitations. They generally require large, accurately labelled datasets and substantial computational resources to achieve reliable and generalizable performance [45,48]. Their decision-making processes may also be difficult to interpret, raising concerns regarding transparency, clinician trust, and accountability [49].

Successful clinical implementation therefore requires careful attention to data quality, external validation, interpretability, computational infrastructure, and integration with the pediatric dentist’s professional judgment.

### 3.3. Convolutional Neural Networks and Imaging-Based Architectures (CNNs and IBAs)

Convolutional neural networks (CNNs) are deep-learning architectures designed primarily for image analysis and automated extraction of spatial features from images [50,51,52]. In dental radiographs and clinical photographs, CNN-based systems can identify clinically relevant findings, including carious lesions [11,13,14,15,16], dental plaque [17], teeth and tooth boundaries [9,25], and developmental anomalies such as mesiodens [26]. More advanced imaging architectures, including U-Net, residual networks, vision transformers, and hybrid models, may further improve lesion localization and anatomical segmentation [53,54,55,56]. Nevertheless, their clinical utility depends on robust internal and external validation, adequate and standardized image quality, and interpretation of model outputs within the broader clinical context by trained clinicians [6,12,15,16,30].

Although CNNs remain the backbone of dental image analysis, contemporary research increasingly employs more advanced architectures. U-Net, with its encoder–decoder structure and skip connections, has become the dominant model for segmentation tasks such as tooth boundary delineation and lesion localization [53]. Residual networks (ResNet) introduce identity-based skip connections that enable the training of very deep models without degradation, improving performance in complex classification and detection tasks [54]. More recently, vision transformers (ViT) have emerged as an alternative to convolution-based models by using self-attention mechanisms to capture long-range dependencies, an advantage for panoramic radiographs and CBCT slices where clinically relevant features may be spatially distant [55]. Hybrid architectures that combine convolutional backbones with transformer-based modules are also gaining traction, integrating strong local feature extraction with global contextual reasoning [56]. Together, these imaging-based architectures represent an evolving ecosystem of tools that extend beyond classical CNNs, positioning dental AI to benefit from the rapid advances occurring across the broader field of medical image analysis.

### 3.4. Data Augmentation and Transfer Learning

Data augmentation increases the diversity of limited training datasets by creating realistic variations in existing images, which may reduce overfitting and improve model robustness. Transfer learning allows models previously trained on large image datasets to be adapted for dental applications [57,58,59,60]. Both approaches are particularly useful in pediatric dentistry, where large, well-annotated datasets may be difficult to obtain. Nevertheless, their performance must be confirmed using independent clinical data [6,30].

### 3.5. Explainable and Trustworthy AI

As AI systems become increasingly integrated into dental diagnostics, the need for explainability and trustworthiness has become paramount [61,62,63]. Deep learning models, particularly CNNs and transformer-based architectures, are often criticized for their “black box” nature, where the internal decision-making process is difficult to interpret [64,65]. Explainable AI (XAI) techniques aim to address this limitation by providing visual or quantitative insights into how a model arrives at its predictions. Methods such as saliency maps, Grad-CAM, integrated gradients, and attention visualization highlight the regions of an image that most strongly influenced the model’s decision, enabling clinicians to verify that the system is focusing on anatomically and clinically relevant structures [66,67,68].

Trustworthy AI extends beyond interpretability to include reliability, fairness, robustness, and clinical accountability [69,70,71,72]. In dentistry, trustworthy systems should perform consistently across diverse patient populations, imaging devices, and clinical settings. They also require transparent performance reporting, external validation, and safeguards against algorithmic bias [6,71]. Explainable and trustworthy AI frameworks are therefore essential for promoting clinician confidence, supporting regulatory approval, and ensuring that AI enhances rather than compromises patient care.

This section has outlined the foundational concepts needed to understand the reviewed evidence. The following sections focus on the clinical benefits, limitations, and practical applications of AI in pediatric dentistry.

## 4. Advantages of AI in Pediatric Dentistry

AI offers several advantages:

### 4.1. High Diagnostic Accuracy

Artificial intelligence significantly enhances diagnostic performance in pediatric dentistry, particularly in imaging-based applications such as caries detection and anomaly identification. Deep learning models, especially convolutional neural networks, have demonstrated high diagnostic performance, owing to their ability to detect subtle patterns within radiographic and photographic data that may not be readily apparent during conventional clinical examination [13,14,15,16,25,26].

### 4.2. Reduction in Observer Variability

AI contributes to minimizing inter- and intra-observer variability, thereby improving consistency in diagnosis and treatment planning. This is particularly important in pediatric dentistry, where diagnostic interpretation may vary based on clinician experience and subjective judgment [6].

### 4.3. Enhanced Efficiency and Automation

AI-driven systems facilitate rapid image analysis and automate repetitive tasks, significantly improving workflow efficiency. This allows clinicians to allocate more time to patient-centered care and complex clinical decision-making [7].

### 4.4. Early Disease Detection

AI models enable the detection of early-stage lesions and subtle pathological changes, supporting timely intervention and preventive strategies. Early diagnosis is especially critical in pediatric dentistry to prevent disease progression and preserve dental structures [15,16].

### 4.5. Predictive and Personalized Care

Machine learning algorithms can integrate multiple clinical and behavioral variables to generate predictive insights. This supports risk stratification and personalized treatment planning tailored to individual patient profiles [22,23,24].

### 4.6. Improved Clinical Decision-Making

By combining high diagnostic accuracy with predictive capabilities, AI enhances clinical decision-making processes. Several studies have demonstrated that AI performance in specific diagnostic tasks can be comparable to that of experienced clinicians [11,13].

## 5. Disadvantages and Challenges

Despite its advantages, AI has several limitations:

### 5.1. Limited Generalizability

A major limitation of current AI models is their restricted generalizability, as many are trained on single-center datasets with limited demographic diversity. This raises concerns regarding their applicability across different populations and clinical environments [20,30].

### 5.2. Lack of External Validation

Although many AI systems demonstrate high performance under controlled experimental conditions, their effectiveness in real-world clinical settings remains insufficiently validated. The absence of large-scale, multicenter validation studies limits confidence in their routine use [15,16].

### 5.3. Overreliance on Imaging Data

Most AI applications in dentistry are heavily dependent on imaging data, with limited integration of essential clinical information such as patient history, behavioral factors, and socioeconomic determinants, which are particularly relevant in pediatric care [8,30].

### 5.4. Limited Interpretability (“Black Box” Issue)

Many deep-learning models generate predictions without clearly explaining how the decision was reached. This “black box” nature may reduce clinician trust and make it difficult to identify incorrect or biased recommendations. False-positive outputs may expose children to unnecessary investigations or treatment, whereas false-negative outputs may delay intervention or allow pathology to progress. Because such errors can directly affect treatment planning and clinical outcomes, AI-generated recommendations must be reviewed by a pediatric dentist and correlated with the clinical and radiographic findings before action is taken. In pediatric dentistry, AI outputs should therefore remain subject to professional interpretation and human oversight. Explainable AI methods may improve transparency by showing the clinical features that influenced a prediction; however, the pediatric dentist should retain final responsibility for diagnosis and treatment decisions [6,49,61].

### 5.5. Ethical, Legal, and Regulatory Considerations

The clinical use of AI in pediatric dentistry raises important ethical, legal, and regulatory concerns. Pediatric dental records and images contain sensitive personal information and therefore require secure storage, controlled access, encryption, and protection against unauthorized use and cybersecurity breaches. Before AI is incorporated into diagnosis or treatment planning, parents or legal guardians should receive a clear explanation of the system’s intended role, potential benefits and limitations, data-use implications, and possible influence on clinical decisions, and should provide informed permission. Age-appropriate assent should also be obtained from the child where applicable. Additional safeguards are required when pediatric data are reused for algorithm development or model training, including appropriate ethical approval, secure de-identification, data minimization, and valid parental or guardian authorization.

Algorithmic bias is another important concern, as models trained on unrepresentative datasets may perform inconsistently across demographic, socioeconomic, or geographic groups. Transparency and explainability are therefore essential to enable clinicians to critically assess AI-generated recommendations. Recent risk-based regulatory frameworks, including the European Union Artificial Intelligence Act, emphasize data governance, technical documentation, risk management, human oversight, transparency, and continuous performance monitoring. AI should support rather than replace clinical judgment, with the pediatric dentist remaining responsible for interpreting and verifying its outputs, communicating the final diagnosis and treatment plan, and maintaining accountability for errors or adverse outcomes [6,30,71].

### 5.6. Practical Implementation Challenges

Implementing AI systems in routine pediatric dental practice presents several practical challenges. These include the costs of software acquisition, licensing, maintenance, hardware upgrades, cybersecurity, and technical support. AI tools must also be compatible with existing electronic dental records, radiographic systems, imaging devices, and clinical workflows. Poor interoperability may increase workload rather than improve efficiency.

Successful implementation further requires staff training in the interpretation and limitations of AI-generated outputs, data protection, ethical use, and professional liability. Clinics must also establish procedures for system updates, model recalibration, performance monitoring, and the detection of model drift over time. Additional barriers may include workflow disruption, clinician acceptance, uncertain reimbursement, vendor dependence, and limited access to technical expertise, particularly in low-resource settings.

## 6. Applications of Artificial Intelligence in Pediatric Dentistry

Whereas Section 3 explains the principal AI approaches, this section examines how these methods have been applied to specific diagnostic, preventive, and predictive tasks in pediatric dental practice.

### 6.1. Dental Plaque Detection

Artificial intelligence has demonstrated significant utility in the detection of dental plaque, particularly through the application of deep learning models trained on intraoral images. Convolutional neural networks can accurately identify plaque accumulation on tooth surfaces, enabling objective assessment and improving preventive care strategies in pediatric populations. This application supports early intervention and enhances patient education by providing visual and quantifiable feedback on oral hygiene status [17,20].

### 6.2. Assessing Children’s Oral Health

Machine learning models have been increasingly employed to assess overall oral health status in children by integrating clinical, behavioral, and socioeconomic data. These models can identify key risk factors associated with poor oral health, such as dietary habits, oral hygiene practices, and parental education levels. By analyzing multidimensional datasets, AI systems facilitate early risk prediction and enable personalized preventive strategies tailored to individual patient profiles [23,24].

### 6.3. Mesiodens and Supernumerary Tooth Identification

Deep learning approaches, particularly CNN-based models, have shown high accuracy in detecting mesiodens and other supernumerary teeth in panoramic radiographs. These anomalies, which are relatively common in pediatric populations, can be challenging to diagnose using conventional methods. AI-assisted detection improves diagnostic sensitivity and reduces the likelihood of missed or delayed diagnoses, thereby supporting timely clinical management [26].

### 6.4. Detection of Early Childhood Caries (ECC)

Artificial intelligence has been extensively applied in the detection of ECC, one of the most prevalent conditions in pediatric dentistry. Deep learning models demonstrate high sensitivity and specificity in identifying both cavitated and non-cavitated lesions, often achieving strong area under the curve (AUC) values. Importantly, several studies have reported diagnostic performance comparable to that of experienced clinicians, highlighting the potential of AI as a supportive diagnostic tool in clinical practice [13,15,16].

### 6.5. Fissure Sealant Categorization

AI systems have been developed to classify fissure sealants and evaluate their integrity over time using dental imaging data. These models can differentiate between intact, partially lost, and failed sealants, facilitating longitudinal monitoring and improving preventive care outcomes. Such applications are particularly valuable in pediatric dentistry, where fissure sealants play a critical role in caries prevention [20].

### 6.6. Chronological Age Assessment

Deep learning models have shown strong performance in estimating chronological age based on dental and skeletal development. By analyzing radiographic features, these systems can provide accurate age predictions with a mean error often less than one year. This capability is useful in orthodontic planning, growth assessment, and forensic applications, particularly in pediatric populations where developmental changes occur rapidly [27,29].

### 6.7. Detection of Primary and Young Permanent Teeth

AI models, particularly those based on object detection and segmentation algorithms, have demonstrated high accuracy in identifying and numbering primary and young permanent teeth in pediatric radiographs. With F1 scores frequently exceeding 0.90, these systems support automated dental charting and assist clinicians in evaluating mixed dentition stages, thereby improving diagnostic efficiency and treatment planning [9,25].

### 6.8. Ectopic Eruption of First Permanent Molar

Artificial intelligence has also been applied to the early detection of ectopic eruption of the first permanent molar, a condition that may lead to malocclusion if not identified promptly. By analyzing radiographic patterns, AI models can detect abnormal eruption pathways, enabling early orthodontic intervention and reducing the risk of long-term complications [20].

### 6.9. Detection of Dental Anomalies

AI-driven systems are increasingly capable of identifying a wide range of dental anomalies, including hypodontia, taurodontism, and structural abnormalities. Through advanced image analysis, these models provide consistent and reliable detection, supporting clinicians in diagnosing complex conditions that may otherwise be overlooked. This contributes to improved diagnostic accuracy and more comprehensive treatment planning in pediatric dental care [20,26,30].

Artificial intelligence is progressively reshaping pediatric dentistry by enabling a wide spectrum of applications spanning early diagnosis, risk prediction, and treatment planning. Across the reviewed studies, AI models, particularly those based on deep learning, demonstrate consistently high performance in detecting dental caries, identifying anomalies, assessing developmental stages, and supporting preventive strategies. Although variations exist in study design, datasets, and evaluation metrics, the collective evidence highlights a clear trend toward the integration of data-driven approaches into pediatric dental practice. These advancements underscore the potential of AI as a complementary tool to enhance clinical decision-making and optimize patient outcomes. Representative studies illustrating these applications, along with their methodologies and key outcomes, are summarized in Table 1.

The chronological distribution of studies highlights the progressive evolution of AI in pediatric dentistry, beginning with early applications focused primarily on image-based caries detection and automated tooth identification (2018–2020), followed by the emergence of machine learning models for risk prediction and preventive assessment (2021–2022), and more recently, advanced deep learning approaches for clinical validation, skeletal growth prediction, and dental age estimation (2024–2025). Collectively, the reviewed studies demonstrate consistently high diagnostic and predictive performance; however, substantial heterogeneity remains in dataset composition, AI architectures, outcome measures, and validation methodologies. These variations emphasize the ongoing need for standardized reporting protocols, larger multicenter datasets, and prospective clinical validation studies to facilitate reliable translation of AI technologies into routine pediatric dental practice.

## 7. Discussion

The present review highlights the rapidly expanding role of AI in pediatric dentistry, particularly in diagnostic imaging and predictive analytics. The majority of current evidence demonstrates that AI-based models, especially deep learning algorithms, can achieve high levels of diagnostic accuracy in tasks such as caries detection, anomaly identification, and dental age estimation. In several instances, the performance of these models has been reported to be comparable to that of experienced clinicians, suggesting that AI has the potential to serve as a valuable adjunct in clinical decision-making [13,14,15,16,25,26,27,28,29,30]. These findings are particularly relevant in pediatric dentistry, where early diagnosis and preventive care are critical for long-term oral health outcomes [11,20].

However, the strength of this evidence should be interpreted cautiously. Many published studies have used relatively small, retrospective, and single-center datasets, often composed of carefully selected, high-quality images. Such datasets may not adequately represent differences in age, demographic background, disease prevalence, socioeconomic conditions, imaging devices, or acquisition protocols. Class imbalance, inconsistent reference standards, and incomplete reporting of data partitioning may also inflate model performance. Furthermore, inadequate separation of patients between training and testing datasets may introduce data leakage, while repeated model optimization using the same internal datasets increases the risk of overfitting. These methodological limitations reduce reproducibility, generalizability, and confidence in the reported performance of AI models.

Despite these promising results, a considerable gap remains between technical performance and real-world clinical implementation. Most of the studies included in the current literature are retrospective in nature and are conducted under controlled experimental conditions, often using curated datasets with high-quality images. While such conditions facilitate model development and optimization, they do not adequately reflect the variability encountered in routine clinical practice, including differences in imaging quality, patient cooperation, and operator-dependent factors [15,30]. As a result, the generalizability and robustness of many AI models remain uncertain when applied in diverse clinical environments.

Another important limitation is the predominant reliance on imaging data. Although radiographs and intraoral images provide valuable diagnostic information, pediatric dental care is inherently multifactorial, requiring consideration of clinical examination findings, behavioral factors, dietary habits, and socioeconomic determinants. The limited integration of these variables into current AI models restricts their ability to provide comprehensive and context–aware clinical support. Future research should therefore focus on the development of multimodal AI systems capable of combining imaging data with clinical and patient-specific information to enhance predictive accuracy and clinical relevance [22,23,24,40].

External validation remains limited because many AI models have been assessed only using the datasets from which they were developed. Although internal validation and cross-validation can estimate performance within a given dataset, they do not establish whether a model will perform reliably across different populations, institutions, imaging devices, or clinical workflows. To date, relatively few systems have been evaluated in independent, multicenter, or prospective cohorts. Large-scale external validation and prospective clinical studies are therefore needed to determine the robustness, reproducibility, and generalizability of AI systems in pediatric dentistry [15,16].

Although some AI systems can independently generate predictions for narrowly defined tasks, such as caries detection or tooth identification, this should not be considered autonomous clinical diagnosis. Current evidence does not demonstrate that these systems can independently integrate clinical examination findings, patient history, behavioral factors, developmental stage, and radiographic information. Therefore, current AI technologies are not sufficiently validated for autonomous diagnosis in routine pediatric dental practice and should remain clinician-supervised decision-support tools [15,16,20,30].

Importantly, high accuracy, sensitivity, specificity, or area-under-the-curve values indicate technical performance but do not necessarily demonstrate clinical usefulness. These measures do not establish whether AI improves diagnostic decisions, patient outcomes, workflow efficiency, cost-effectiveness, or safety in routine practice. Future studies should therefore compare AI-assisted and unassisted clinicians under real-world conditions and evaluate clinically meaningful outcomes rather than relying exclusively on retrospective performance metrics. Prospective studies should also evaluate and report the clinical consequences of false-positive and false-negative predictions, including unnecessary treatment, delayed care, missed pathology, and inappropriate treatment planning.

AI should not be introduced solely because it represents a technological advancement. Its adoption should be justified by measurable added value over existing clinical practice, such as improved diagnostic consistency, reduced observer variability, faster image assessment, earlier disease detection, enhanced risk stratification, or improved access to specialist support. These potential benefits must be confirmed through prospective studies evaluating clinical effectiveness, patient outcomes, workflow impact, cost-effectiveness, and safety before routine implementation [6,7,20,30].

In addition to technical and methodological limitations, ethical and regulatory considerations are central to the safe implementation of AI in pediatric dentistry. The use of sensitive pediatric data requires robust privacy protection, cybersecurity safeguards, parental or guardian permission, and age-appropriate assent where applicable. Unrepresentative training datasets may also introduce algorithmic bias and unequal diagnostic performance across patient groups. Explainability, transparency, human oversight, and clearly defined professional accountability are therefore essential. Recent risk-based regulatory frameworks emphasize data governance, technical documentation, continuous risk assessment, and post-deployment performance monitoring for healthcare AI systems.

Practical implementation also extends beyond regulatory approval. Dental practices must consider software and hardware costs, compatibility with existing clinical and imaging systems, staff training, technical support, workflow integration, reimbursement, and periodic model updating. AI systems also require stable, secure, and regularly updated computing infrastructure to protect patient confidentiality and maintain reliable clinical performance. Continuous monitoring is particularly important because model performance may decline when patient populations, imaging devices, or clinical practices differ from those used during development. These requirements may create substantial barriers for smaller clinics and healthcare systems with limited digital infrastructure [6,8,30].

From a clinical perspective, the integration of AI into routine pediatric dental practice should be approached cautiously. Current AI systems should be regarded as clinical decision-support tools rather than autonomous diagnostic systems or replacements for professional expertise. Their outputs should be interpreted alongside the clinical examination, patient history, radiographic findings, behavioral factors, and the pediatric dentist’s professional judgment. Such interpretation should be led by pediatric dentists with direct patient-care experience and, where appropriate, supported by clinical educators to ensure that AI-assisted decisions remain evidence-based, developmentally appropriate, and patient-centered. The child’s behavioral and communication needs, family context, and clinician–patient relationship must remain central to care. The final responsibility for diagnosis and treatment planning should remain with the clinician. Successful implementation will also require user-friendly interfaces, clinician training, technical support, and seamless integration into existing clinical workflows [11,14,25].

Future research directions should prioritize prospective clinical trials to evaluate the real-world effectiveness of AI systems, as well as the incorporation of multimodal data to improve model performance. Additionally, efforts should be made to standardize reporting guidelines, evaluation metrics, and benchmarking datasets to facilitate comparison across studies. Addressing these challenges will be essential to bridge the gap between experimental research and clinical application, ultimately enabling the safe and effective integration of AI into pediatric dentistry [15,16,30].

## 8. Limitations

This review has several limitations that should be considered when interpreting its findings. Although a structured literature search and predefined eligibility criteria were used, the review was narrative rather than systematic. Consequently, study selection remained partly dependent on author judgment and may have been subject to selection bias or incomplete literature coverage. In addition, no formal risk-of-bias assessment or quantitative meta-analysis was performed, limiting the ability to draw definitive conclusions regarding the comparative performance of different AI models across studies [7,8,30].

The included studies also showed considerable heterogeneity in their designs, dataset characteristics, AI architectures, reference standards, validation approaches, and performance metrics. This variability restricted direct comparison between studies and reduced the generalizability of the conclusions. Furthermore, inconsistencies in the reporting of AI studies in dentistry may have affected the transparency, reproducibility, and interpretation of the available evidence [6,7,8,30].

Finally, artificial intelligence in pediatric dentistry is a rapidly evolving field, with new models, datasets, and validation studies continuing to emerge. Although the literature search was updated to 6 May 2026, subsequently published developments may not have been captured. The findings should therefore be interpreted as a structured contemporary overview rather than a comprehensive or definitive synthesis of all available evidence [15,16,29].

## 9. Conclusions

Artificial intelligence is increasingly shaping the future of pediatric dentistry by introducing data-driven approaches to diagnosis, risk assessment, and treatment planning. While current evidence demonstrates considerable potential, the true value of AI lies in its ability to complement clinical expertise rather than replace it. The transition from experimental models to clinically applicable tools will depend on the development of robust, generalizable systems that are seamlessly integrated into routine practice.

Moving forward, emphasis should be placed on clinically relevant validation, interdisciplinary collaboration, and the design of user-centered AI systems that align with the specific needs of pediatric dental care. Addressing these priorities will be essential to translate technological advancements into meaningful improvements in patient outcomes.

## Figures and Tables

**Table 1:** Summary of representative studies on AI applications in pediatric dentistry, including study design or dataset, AI model, and key outcomes, arranged chronologically.

No.	Author (Year)	Study Design/Dataset	Application	AI Model	Key Outcomes
1	Lee et al. [11] (2018)	Radiographic dataset	Caries detection and diagnosis	CNN	Demonstrated diagnostic feasibility of CNN-based caries detection
2	Chen et al. [9] (2019)	Dental periapical films	Tooth detection and numbering	Deep learning object detection	Accurate automatic tooth detection and numbering
3	Hwang et al. [12] (2019)	Narrative overview	Deep learning in dentistry	Deep learning	Summarized major dental DL applications and limitations
4	Karhade et al. [22] (2021)	Clinical dataset	ECC classification	AutoML	Automated classifier for early childhood caries
5	Lian et al. [13] (2021)	Image-based dataset	Caries detection and classification	Deep learning	Supported automated caries detection and lesion classification
6	Kim et al. [26] (2022)	Pediatric panoramic radiographs	Mesiodens identification	Deep learning	Accurate identification using automatic maxillary anterior region estimation
7	Kaya et al. [25] (2022)	Pediatric panoramic radiographs	Permanent tooth germ detection	Deep learning	AP = 94.16%; F1 score = 0.90
8	Szabó et al. [15] (2024)	Intraoral bitewing and periapical radiographs	Caries diagnosis validation	AI application	Validated AI-assisted caries diagnosis on intraoral radiographs
9	Sadegh–Zadeh et al. [23] (2024)	Clinical and questionnaire data from children	Dental health risk assessment	ML models	Identified oral hygiene, sugary diet, and fluoride exposure as key factors
10	Mohammed et al. [28] (2024)	Dental patient imaging data	Skeletal growth prediction	CNN	Applied CNN-based methods for skeletal growth prediction
11	Tan et al. [16] (2025)	Bitewing radiographs of primary molars	Caries detection	Deep learning object detectors	Applied object detectors to detect caries in primary molars
12	Chen et al. [21] (2025)	Children aged 9 years	First molar caries prediction	ML model	Predicted caries risk in first molars of children
13	Hasan et al. [24] (2025)	Bangladesh pediatric dataset	ECC risk prediction	ML approaches	Predicted early childhood caries risk using ML
14	Balel et al. [29] (2025)	Panoramic radiographs	Dental age estimation	Deep learning	Developed and evaluated DL-based age estimation using the Demirjian method

CNN = convolutional neural network; DL = deep learning; ML = machine learning; AutoML = automated machine learning; AP = average precision; ECC = early childhood caries.

## Data Availability

No new data were created or analyzed in this study. Data sharing is not applicable to this article.

## Abbreviations

The following abbreviations are used in this manuscript:

AI	Artificial Intelligence.
ML	Machine Learning
DL	Deep Learning
ANN	Artificial Neural Network
DNN	Deep Neural Network
CNNs	Convolutional Neural Networks
XAI	Explainable Artificial Intelligence
RL	Reinforcement Learning
MLP	Multilayer Perceptron
ViT	Vision Transformer
Grad-CAM	Gradient-weighted Class Activation Mapping
ECC	Early Childhood Caries
AP	Average Precision
AUC	Area Under the Curve
ROC	Receiver Operating Characteristic
IT	Information Technology
U-Net	U-shaped Convolutional Network
ResNet	Residual Neural Network
CNN-Based Object Detection	Convolutional Neural Network-Based Object Detection
AutoML	Automated Machine Learning
3D	Three-Dimensional
DL-Based	Deep Learning-Based
AI-Assisted	Artificial Intelligence-Assisted
CBCT	Cone-Beam Computed Tomography
